# Alcohol and red wine consumption, but not fruit, vegetables, fish or dairy products, are associated with less endothelial dysfunction and less low-grade inflammation: the Hoorn Study

**DOI:** 10.1007/s00394-017-1420-4

**Published:** 2017-03-27

**Authors:** B. C. T. van Bussel, R. M. A. Henry, C. G. Schalkwijk, J. M. Dekker, G. Nijpels, E. J. M. Feskens, C. D. A. Stehouwer

**Affiliations:** 1 Department of Medicine, Maastricht University Medical Centre + (MUMC+), Maastricht, The Netherlands; 2 School of Nutrition, and Translational Research in Metabolism (NUTRIM), MUMC+, Maastricht, The Netherlands; 3 Top Institute Food and Nutrition (TIFN), Wageningen, The Netherlands; 4 Cardiovascular Research Institute Maastricht (CARIM), MUMC+, Maastricht, The Netherlands; 5 The EMGO Institute for Health and Care Research (EMGO), Vrije Universiteit Medical Centre (VUMC), Amsterdam, The Netherlands; 6 Department of Epidemiology and Biostatistics, VUMC, Amsterdam, The Netherlands; 7 Department of General Practice, VUMC, Amsterdam, The Netherlands; 8 Division of Human Nutrition, Wageningen University, Wageningen, The Netherlands; 9 Department of Medicine, Maastricht University Medical Centre, Prof. Debyelaan 25, Maastricht, HX 6229 The Netherlands

**Keywords:** Diet, Endothelial dysfunction, Inflammation, Elderly, Red wine

## Abstract

**Purpose:**

Endothelial dysfunction and low-grade inflammation are key phenomena in the pathobiology of cardiovascular disease (CVD). Their dietary modification might explain the observed reduction in CVD that has been associated with a healthy diet rich in fruit, vegetables and fish, low in dairy products and with moderate alcohol and red wine consumption. We investigated the associations between the above food groups and endothelial dysfunction and low-grade inflammation in a population-based cohort of Dutch elderly individuals.

**Methods:**

Diet was measured by food frequency questionnaire (*n* = 801; women = 399; age 68.5 ± 7.2 years). Endothelial dysfunction was determined (1) by combining von Willebrand factor, and soluble intercellular adhesion molecule 1 (sICAM-1), vascular cell adhesion molecule 1, endothelial selectin and thrombomodulin, using Z-scores, into a biomarker score and (2) by flow-mediated vasodilation (FMD), and low-grade inflammation by combining C-reactive protein, serum amyloid A, interleukin 6, interleukin 8, tumour necrosis factor α and sICAM-1 into a biomarker score, with smaller FMD and higher scores representing more dysfunction and inflammation, respectively. We used linear regression analyses to adjust associations for sex, age, energy, glucose metabolism, body mass index, smoking, prior CVD, educational level, physical activity and each of the other food groups.

**Results:**

Moderate [β (95% CI) −0.13 (−0.33; 0.07)] and high [−0.22 (−0.45; −0.003)] alcohol consumption, and red wine [−0.16 (−0.30; −0.01)] consumption, but none of the other food groups, were associated with a lower endothelial dysfunction biomarker score and a greater FMD. The associations for FMD were, however, not statistically significant. Only red wine consumption was associated with a lower low-grade inflammation biomarker score [−0.18 (−0.33; −0.04)].

**Conclusions:**

Alcohol and red wine consumption may favourably influence processes involved in atherothrombosis.

**Electronic supplementary material:**

The online version of this article (doi:10.1007/s00394-017-1420-4) contains supplementary material, which is available to authorized users.

## Introduction

A healthy diet, rich in fruit, vegetables and fish, low in dairy products and with moderate alcohol and red wine consumption, is associated with a reduced incidence of cardiovascular disease (CVD) [1]. Endothelial dysfunction and low-grade inflammation are key phenomena in the pathobiology of CVD [2] and seem to be influenced by dietary intake to the extent that a healthy diet is associated with less endothelial dysfunction and less low-grade inflammation [3–5]. A healthy diet, therefore, could potentially reduce the incidence of CVD by acting favourably upon both endothelial dysfunction and low-grade inflammation.

Several of the previous population-based studies on the association between diet and biomarkers of endothelial dysfunction and biomarkers of low-grade inflammation focused on diet scores that included fish, fruit, vegetables, dairy products, and alcohol and red wine, whereas others investigated some of these food components separately [3–8]. None of the previous studies, however, examined each of these food components of a healthy diet in relation to each other [9]. Notably, population-based data on the association between diet and flow-mediated vasodilation (FMD) of the brachial artery, an estimate of endothelium-dependent vasodilator function, are scarce [10, 11].

The consumption of red wine has been shown to act favourably upon endothelial dysfunction and low-grade inflammation [12]. It remains, however, unclear to what extent alcohol itself (i.e. ethanol) or any additional nutrient components such as polyphenols and flavonoids determine the association between red wine and endothelial dysfunction and low-grade inflammation [13].

We therefore examined, in order to investigate in detail the associations between a healthy diet and endothelial dysfunction and low-grade inflammation, the associations between, on the one hand, fruit, vegetables, fish, dairy products, alcohol and red wine consumption and, on the other, five biomarkers of endothelial dysfunction and FMD, and six biomarkers of low-grade inflammation. In addition, we investigated whether any such associations were independent of each of the other food components of a healthy diet and lifestyle risk factors. Although the results will focus on any independent associations, we systematically report on each of these food groups in a large population-based cohort study of elderly individuals.

## Methods

### Study population

For the present study, data were used from the 2000 Hoorn Study follow-up examination, previously described elsewhere [14–16]. Of the original 822 participants, 20 were without dietary and biomarker data and one was without all covariate data (by participating only partially in the first study visit only) [14]. Of the remaining 801 participants, 63 were without biomarker data [16] and 158 were without FMD data [15]. Thus, for the present analyses full data on diet and biomarkers were available in 738 participants [16] and full data on diet and FMD were available in 643 participants [15]. The local ethics committee approved the study and all participants gave their written informed consent.

### Dietary intake

Diet was assessed by a validated self-administered food frequency questionnaire (FFQ) originally developed for Dutch participants of the EPIC study [17–19]. The FFQ queried participants to report habitual diet over the previous year. Energy and ethanol intake were calculated with the help of the extended Dutch food composition database from 1996. We assessed fruit (g/d), vegetables (g/d), fish (g/d) and dairy product (g/d) consumption, which were categorized into tertiles, and alcohol (g/d) consumption, which was categorized as follows: nonconsumers (no consumption of alcohol-containing beverages); moderate consumers (men: ethanol intake >0 and ≤20 g/d; women: ethanol intake >0 and ≤10 g/d) and high consumers (men: ethanol intake >20 g/d; women: ethanol intake >10 g/d).

Red wine was dichotomized into nonconsumers (no consumption of red wine) and consumers (red wine consumption; median (IQR) 28.6 (5.5–71.4) dL/d) and associations for alcohol and red wine were, additionally, mutually adjusted for each other.

### Biomarkers of endothelial dysfunction and low-grade inflammation

Biomarkers were quantified in serum samples by a multi-array detection system based on electrochemiluminescence technology (SECTOR Imager 2400, Meso Scale Discovery, Gaithersburg, MD, USA). sICAM-1, sVCAM-1, sE-selectin and sTM were determined as biomarkers of endothelial dysfunction, and CRP, SAA, IL-6, IL-8, TNF-α and sICAM-1 were determined as biomarkers of low-grade inflammation. All serum samples were measured on a single production lot of multi-array plates and in duplication on separate designated wells on the same array plate. Intra- and inter-assay CV were for sICAM-1, 2.4 and 4.9%; for sVCAM-1, 2.8 and 5.6%; for sE-selectin, 2.6 and 6.7%; for sTM, 2.1 and 6.9%; for CRP, 2.8 and 4.0%; for SAA, 2.7 and 11.6%; for IL-6, 5.6 and 13.0%; for IL-8, 5.6 and 12.2% and for TNF-α, 3.9 and 8.8%, respectively. In addition, von Willebrand factor (vWf), a biomarker of endothelial dysfunction, was determined in citrated plasma by means of ELISA as described elsewhere [16]. Intra- and inter-assay CV were for vWf, 3.4 and 7.9%, respectively.

### Flow-mediated vasodilation

FMD was measured in accordance with international guidelines [20, 21]. This method allows for the determination of the peak arterial vasodilatory response (maximum diameter) after cuff release [15], which reflects endothelium-dependent vasodilation [22]. Briefly, baseline diameter (mean of three measurements) was assessed with the use of an ultrasound scanner equipped with a 7.5 MHz linear probe (350 series, Pie Medical, Maastricht, The Netherlands) that was connected to a PC equipped with vessel wall movement detection software and an acquisition system (Wall Track System, Pie Medical, Maastricht, The Netherlands). Baseline peak flow velocity (mean of two measurements) was determined by pulsed-wave Doppler from a sample volume in the centre of the artery at a 60° angle. A pressure cuff, placed on the forearm, was then automatically inflated and kept constant at supra-systolic pressure (SBP) (brachial SBP + 100 mmHg) in order to induce forearm ischaemia. After 5 min, the cuff was released leading to an increase in blood flow and hereby in shear stress, which served as the stimulus for FMD response. Maximum peak flow velocity was measured within 15 s after the cuff release, and measures of arterial diameter were subsequently measured at 45, 90, 180 and 300 s.

In some individuals, measures of brachial diameter were missing at one (*n* = 70), two (*n* = 21) or three (*n* = 10) out of the four post-cuff release time points. For these individuals, we imputed the missing post-cuff release diameter(s) with a longitudinal regression method [23]. Specifically, we regressed the repeated post-cuff release measures of diameter available on baseline diameter and time with the use of generalized estimating equations. Because the relationship between time and diameter is not linear, time was treated as a categorical (dummy) variable to approximate the FMD response curve by estimation of the predicted diameter at each exact time point (i.e. at 45, 90,180 or 300 s after cuff release) based on the observed baseline and post-cuff release measures obtained [24]. The predicted values hereby estimated were used to replace the missing value(s). FMD (peak arterial vasodilatory response, i.e. change in mm) was then calculated as maximum post-occlusion diameter minus baseline diameter and was used in the analyses as main dependent variable.

### Other variables

Health status, medical history, glucose metabolism status, medication use, educational level, physical activity and smoking habits were assessed by questionnaire. Height, weight, serum creatinine, albuminuria, total and HDL cholesterol, blood pressure, BMI (kg/m^2^) and prior CVD were determined as described elsewhere [14–16]. Educational level was classified into six categories: primary education only; low level of secondary education; high level of secondary education; and tertiary education, either a bachelor degree, master degree or PhD. Physical activity was expressed in metabolic equivalents (METs) (hour/wk), based on the amount of daily activities, including sports, bicycling, gardening, walking, doing odd jobs and housekeeping. Smoking behaviour was dichotomized into current smokers (15.1%) and nonsmokers, which included both previous smokers (45.5%) and never smokers (39.4%). Estimated glomerular filtration rate (eGFR) (mL/min/1.73 m^2^) was calculated according to Levey’s [Chronic Kidney Disease Epidemiology Collaboration] formula [25].

### Statistical analyses

Data analyses were performed with SPSS (Statistical Package for Social Sciences, version 20, IBM Corp., USA). Data were presented as mean (± SD), as median (interquartile range) for skewed variables or as percentages and as regression coefficients (β) with their 95% confidence intervals (95% CI). Variables with a skewed distribution were natural log-transformed in order to meet normality criteria (serum triglycerides, METs, CRP, SAA, IL-6 and IL-8).

#### Endothelial dysfunction

For reasons of statistical efficiency and to reduce the influence of the biological variability of each measure, a biomarker score for endothelial dysfunction was constructed using Z-scores as previously described elsewhere [16, 26–28]. The endothelial dysfunction biomarker score consisted of vWf, sE-selectin, sTM, sVCAM-1 and sICAM-1, with a higher score indicating worse function.

With regard to FMD, the peak arterial vasodilatory response (i.e. the change (in mm)) was used as outcome variable (lower FMD indicating worse function).

#### Low-grade inflammation

The low-grade inflammation biomarker score consisted of CRP, SAA, IL-6, IL-8, TNF-α and sICAM-1 (sICAM-1 was included in both biomarker scores as it is expressed by both monocytes and the endothelium) [16, 26–28], with a higher score indicating more inflammation.

#### Statistical models

Firstly, we used linear regression analyses to investigate the cross-sectional associations between, on the one hand, tertiles of fruit, vegetables, fish and dairy product consumption and categories of alcohol consumption and, on the other hand, the endothelial dysfunction biomarker score and FMD. Secondly, we investigated the cross-sectional associations between the above food groups and the low-grade inflammation biomarker score. These analyses were adjusted for sex, age, glucose metabolism status and energy intake. For FMD, the analyses were additionally adjusted for baseline diameter and flow increase (models 1). These associations were then additionally adjusted for BMI, current smoking, prior CVD, educational level and physical activity (models 2), and for each of the other food components of a healthy diet (i.e. fruit, vegetables, fish, dairy products and/or alcohol) (models 3). Thirdly, we repeated the above analyses for red wine consumption (models 1 to 3). Next, the associations for alcohol (i.e. ethanol) and red wine consumption were mutually adjusted for each other. Finally, the presence of clinical disease may modify any effect of diet on CVD [28, 29]. In particular, it has been suggested that the consumption of red wine may be favourable in CVD [13], whereas the consumption of dairy products may have adverse effects [30]. We used interaction terms between clinical disease and food consumption to investigate any such effects. In the present study, clinical disease was defined as the presence of either CVD (*n* = 242), type 2 diabetes mellitus (*n* = 137) or both (*n* = 184).

A two-sided *P*-value < 0.05 and for interaction < 0.10 was considered statistically significant.

## Results

The results section focuses on the independent associations between diet and endothelial dysfunction and low-grade inflammation (i.e. here alcohol and red wine consumption only). Nevertheless, we systematically report results on each of the food groups in the supplemental material and tables.

### Study population

Ninety-one percent (*n* = 729) of the 801 participants were >60 (range 50–87) years of age. With regard to the consumption of alcohol, 139 individuals were classified as nonconsumers (34 men and 105 women: no consumption of alcohol-containing beverages at all), 414 were classified as moderate consumers (237 men: ethanol intake >0 and ≤ 0 g/d; 177 women: ethanol intake >0 and ≤10 g/d), and 248 were classified as high consumers (131 men: ethanol intake >20 g/d; 117 women: ethanol intake >10 g/d).

Age, the ratio of women to men, the prevalence of type 2 diabetes mellitus, a low-educational level and the use of anti-hypertensive medication decreased over the categories of alcohol consumption. In contrast, current smoking, physical activity, estimated glomerular filtration rate (eGFR) and HDL cholesterol, and the intake of energy, red wine, vegetables and fish increased over the categories of alcohol consumption (Table 1).

**Table 1:** Population characteristics, diet, endothelial dysfunction and low-grade inflammation according to categories of alcohol consumption

	Categories of alcohol consumption			P-trend
	None	Moderate	High	
Population characteristics				
Age, year	70.7 ± 7.1	68.8 ± 7.2	66.8 ± 6.9	<0.001
Sex, Men/Women	34/105	237/177	131/117	<0.001
Glucose metabolism status*				
Normal glucose metabolism, %	23.9	40.4	36.9	
Impaired glucose metabolism, %	18.1	23.8	24.1	
Diabetes mellitus type 2, %	58.0	35.8	39.0	0.006
Body mass index, kg/m^2^	28.1 ± 5.0	27.5 ± 4.1	27.8 ± 3.8	0.591
Current smoking, %	12.9	13.3	19.4	0.048
Prior cardiovascular disease, %	62.8	52.2	52.7	0.105
Low-educational level, %	41.3	26.5	19.8	<0.001
Physical activity, METs hour/week	49 [21–99]	81 [47–129]	85 [51–132]	<0.001
Systolic blood pressure, mmHg	142 ± 22	142 ± 20	142 ± 20	0.651
Diastolic blood pressure, mmHg	83 ± 11	83 ± 11	84 ± 11	0.378
Anti-hypertensive medication, %	51.8	37.0	35.9	0.006
Total cholesterol, mmol/L	5.7 ± 1.1	5.6 ± 1.1	5.9 ± 0.9	0.051
HDL cholesterol, mmol/L	1.3 ± 0.4	1.3 ± 0.4	1.5 ± 0.4	<0.001
LDL cholesterol, mmol/L	3.6 ± 1.0	3.6 ± 0.9	3.6 ± 0.8	0.585
Triglycerides, mmol/L	1.5 [1.1–2.2]	1.3 [1.0–1.8]	1.3 [1.0–2.0]	0.838
Lipid-lowering medication, %	18.0	15.9	17.7	0.931
Albuminuria [albumin/creatinine ratio > 2 (mg/mmol)], %	15.1	16.3	13.8	0.624
Serum creatinine, µmol/L	91.6 ± 17.1	96.4 ± 17.7	95.6 ± 16.1	0.083
eGFR, mL/min/1.73 m^2^	60.9 ± 12.4	63.5 ± 11.2	64.2 ± 11.1	0.014
Diet				
Energy intake, kJ/d	7063 ± 2323	8032 ± 2065	9070 ± 3709	<0.001
Ethanol, g/d	0.0 [0.0–0.0]	3.5 [1.0-8.1]	28.7 [20.1–40.0]	By definition
Red wine, dL/d	0.0 [0.0–0.0]	0.0 [0.0–14.3]	12.1 [0.0–85.7]	<0.001
Vegetables, g/d	121 ± 50	125 ± 49	136 ± 61	0.005
Fruit, g/d	302 ± 177	288 ± 168	271 ± 164	0.068
Fish, g/d	4.4 [0.0-14.9]	8.8 [2.8–16.8]	13.2 [4.3–18.0]	<0.001
Dairy products, g/d	450 ± 311	470 ± 270	413 ± 210	0.072
Endothelial dysfunction				
Endothelial dysfunction score, SD	0.15 ± 0.66	0.002 ± 0.66	−0.08 ± 0.59	0.002
von Willebrand factor, %	167 ± 53	155 ± 60	148 ± 46	0.002
Soluble endothelial selectin, µg/L	20 ± 9	19 ± 8	20 ± 8	0.426
Soluble thrombomodulin, µg/L	3.6 ± 1.0	3.6 ± 0.9	3.4 ± 0.9	0.012
Soluble vascular cell adhesion molecule 1, µg/L	437 ± 102	417 ± 103	398 ± 87	<0.001
Soluble intercellular adhesion molecule 1, µg/L	275 ± 68	261 ± 64	261 ± 62	0.115
Flow-mediated vasodilation				
Baseline diameter, mm	4.46 ± 0.75	4.71 ± 0.73	4.72 ± 0.76	0.014
Peak diameter after cuff release, mm	4.60 ± 0.75	4.87 ± 0.71	4.92 ± .0.75	0.001
Peak diameter change after cuff release, mm	0.14 ± 0.11	0.16 ± 0.17	0.20 ± 0.18	0.001
Low-grade inflammation				
Low-grade inflammation score, SD	0.13 ± 0.61	-0.02 ± 0.61	−0.03 ± 0.65	0.051
C-reactive protein, mg/L	3.1 [1.5–5.9]	2.3 [1.2–4.9]	2.4 [1.1–4.3]	0.040
Serum amyloid A, mg/L	2.6 [1.3–3.5]	1.7 [1.0–3.2]	1.7 [1.0–3.2]	0.027
Interleukin-6, ng/L	1.7 [1.1–2.5]	1.5 [1.1–2.2]	1.4 [1.0–2.4]	0.597
Interleukin-8, ng/L	15.5 [11.8–20.4]	11.0 [14.0–18.1]	13.9 [10.9–19.3]	0.953
Tumour necrosis factor α, ng/L	9.2 ± 2.5	8.8 ± 3.0	8.8 ± 3.1	0.361
Soluble intercellular adhesion molecule 1, µg/L	275 ± 68	261 ± 64	261 ± 62	0.115

Data are means ± SD, median [interquartile range] or percentages, as appropriate, according to the following categories of alcohol consumption: nonconsumers (*n* = 139); moderate consumers (men: ethanol intake 0–20 g/d; women: ethanol intake 0–10 g/d; *n* = 414); and high consumers (men: ethanol intake > 20 g/d; women: ethanol intake > 10 g/d; n = 248); P-trend by linear regression analysis for continuous variables and by Chi-square test for proportions

*METs* metabolic equivalents; *HDL* high-density lipoprotein; *LDL* low-density lipoprotein; *eGFR* estimated glomerular filtration rate according to Levey’s [Chronic Kidney Disease Epidemiology Collaboration (CKD-epi)] formula for the following four strata (only Caucasians present in the Hoorn study population); (1) women, serum creatinine ≤ 62 µmol/L: eGFR = 144 × (serum creatinine/0.7)^−0.329^ × (0.993)^Age^ ; (2) women, serum creatinine > 62 µmol/L: eGFR = 144 × (serum creatinine/0.7)^−1.209^ × (0.993)^Age^ ; (3) men, serum creatinine ≤ 80 µmol/L: eGFR = 141 × (serum creatinine/0.9)^−0.411^ × (0.993)^Age^ ; (4) men, serum creatinine > 80 µmol/L: eGFR = 141 × (serum creatinine/0.9)^−1.209^ × (0.993)^Age^ [25]

*Normal glucose metabolism: fasting plasma glucose <6.1 mmol/L and 2-h post-load plasma glucose <7.8 mmol/L; impaired glucose metabolism: impaired fasting plasma glucose between 6.1 and 7.0 mmol/L and impaired glucose tolerance defined as a 2-h post-load plasma glucose between 7.8 and 11.1 mmol/L; diabetes mellitus type 2: fasting plasma glucose >7.0 mmol/L and/or 2-h post-load plasma glucose ≥11.1 mmol/L

As compared to the participants with full data, those without either biomarker (*n* = 63) or FMD (*n* = 158) data had a higher BMI and lower educational level, and used anti-hypertensive medication more frequently (*P*-values <0.05). In addition, those without biomarker data were younger and had type 2 diabetes mellitus more frequently, whereas those without FMD data were older and less physically active, had a lower eGFR and consumed alcohol and red wine less frequently (*P*-values <0.05, other data not shown). The presence of clinical disease in participants with data was 70%.

### Alcohol consumption and endothelial dysfunction

After adjustment for sex, age, glucose metabolism status and energy intake, moderate and high consumers had a lower endothelial dysfunction biomarker score [β (95% CI) −0.16 (−0.37; 0.04) and −0.23 (−0.45; −0.01), respectively (Table 2, model 1)] as compared to nonconsumers (reference group;* P*-trend = 0.051). The results showed a positive association for FMD that was not statistically significant [0.01 (−0.21; 0.23) and 0.16 (−0.08; 0.39), respectively (Table 2, model 1)]. Additional adjustment for BMI, current smoking, prior CVD, educational level, physical activity and each of the other food components of a healthy diet did not materially change the results (Table 2, models 2–3).

**Table 2:** Associations between categories of alcohol and red wine consumption and endothelial dysfunction and low-grade inflammation

Model	Endothelial dysfunction				Low-grade inflammation	
	Biomarker score		Flow-mediated vasodilation		Biomarker score	
	β (95% CI) *P*-value	*P*-trend	β (95% CI) *P*-value	*P*-trend	β (95% CI) *P*-value	*P*-trend
Alcohol						
1						
None	–		–		–	
Moderate	−0.16 (−0.37; 0.04) 0.116		0.01 (−0.21; 0.23) 0.952		−0.12 (−0.32; 0.09) 0.275	
High	0.23 (−0.45;-0.01) 0.040	0.051	0.16 (−0.08; 0.39) 0.200	0.114	−0.07 (−0.30; 0.16) 0.539	0.717
2						
None	–		–		–	
Moderate	−0.13 (−0.33; 0.07) 0.204		−0.02 (−0.24; 0.20) 0.843		−0.08 (−0.28; 0.13) 0.455	
High	−0.23 (−0.45; −0.01) 0.040	0.038	0.17 (−0.06; 0.41) 0.150	0.061	−0.07 (−0.30; 0.15) 0.515	0.594
3						
None	–		–		–	
Moderate	−0.13 (−0.33; 0.07) 0.212		−0.03 (−0.25; 0.19) 0.796		−0.08 (−0.28; 0.13) 0.455	
High	−0.22 (−0.45; −0.003) 0.047	0.047	0.17 (−0.07; 0.41) 0.163	0.073	−0.06 (−0.29; 0.16) 0.574	0.663
4a						
None	–					
Moderate	−0.12 (−0.33; 0.08) 0.226					
High	−0.18 (−0.41; 0.05) 0.122	0.137				
Red wine						
1						
Nonconsumers	–		–		–	
Consumers	−0.21 (−0.35; −0.07) 0.004		0.08 (−0.07; 0.23) 0.291		−0.23 (−0.37; −0.09) 0.002	
2						
Nonconsumers	–		–		–	
Consumers	−0.16 (−0.31; −0.02) 0.028		0.07 (−0.08; 0.23) 0.361		−0.19 (−0.34; −0.04) 0.013	
3						
Nonconsumers	–		–		–	
Consumers	−0.16 (−0.30; −0.01) 0.034		0.07 (−0.09; 0.22) 0.414		−0.18 (−0.33; −0.04) 0.016	
4b						
Nonconsumers	–				–	
Consumers	−0.15 (−0.30; −0.003) 0.045				−0.19 (−0.34; −0.04) 0.012	

Data are standardized regression coefficients, with their 95% confidence intervals, indicating the difference in the endothelial dysfunction biomarker score, flow-mediated vasodilation (FMD) and the low-grade inflammation biomarker score (all in SD) between categories of alcohol consumption, with moderate (men: ethanol intake 0–20 g/d; women: ethanol intake 0–10 g/d) and high consumers (men: ethanol intake >20 g/d; women: ethanol intake >10 g/d) compared to nonconsumers (-, reference group); and between red wine consumers [median (IQR) 28.6 (5.5–71.4) dL/d] and nonconsumers (-, reference group), with *P*-values and P-trend by linear regression analyses

Analyses on biomarker scores include 738 participants and higher values indicate worse function

Analyses on FMD include 643 participants and lower values indicate worse function; to calculate peak diameter change in millimetres multiply by 0.167 (SD)

Model 1: adjusted for sex, age, glucose metabolism status, energy intake and for FMD additionally for baseline diameter and flow increase

Model 2: model 1 additionally adjusted for body mass index, current smoking, prior cardiovascular disease, educational level and physical activity

Model 3: model 2 additionally adjusted for vegetable, fruit, fish and dairy product consumption

Model 4a: model 2 additionally adjusted for red wine (dL/d)

Model 4b: model 2 additionally adjusted for alcohol (g/d)

### Red wine consumption and endothelial dysfunction

After adjustment for sex, age, glucose metabolism status and energy intake, red wine consumers [*n* = 365; median (IQR) intake, 28.6 (5.5–71.4) dL/d] had a lower endothelial dysfunction biomarker score [0.21 (−0.35; −0.07), (Table 2, model 1)] as compared to nonconsumers of red wine (reference group). The results showed a positive association for FMD that was not statistically significant [0.08 (−0.07; 0.23), (Table 2, model 1)]. Additional adjustment for BMI, current smoking, prior CVD, educational level, physical activity and each of the other food components of a healthy diet did not materially change the results (Table 2, models 2–3).

### Mutual adjustment

The association between alcohol consumption and the endothelial dysfunction biomarker score (Table 2, model 2) was attenuated after additional adjustment for red wine consumption (Table 2, model 4a). The association between red wine consumption and the endothelial dysfunction biomarker score (Table 2, model 2) did not materially change after additional adjustment for ethanol (Table 2, model 4b).

### Alcohol consumption, red wine consumption and low-grade inflammation

The consumption of alcohol was not associated with the low-grade inflammation biomarker score. After adjustment for sex, age, glucose metabolism status and energy intake, red wine consumers had a lower low-grade inflammation biomarker score [−0.23 (−0.37; −0.09), (Table 2, model 1)] as compared to nonconsumers of red wine (reference group). Additional adjustment for BMI, current smoking, prior CVD, educational level, physical activity and each of the other food components of a healthy diet did not materially change the results (Table 2, models 2–3). The association between red wine consumption and the low-grade inflammation biomarker score (Table 2, model 2) did not materially change after additional adjustment for ethanol (Table 2, model 4b).

### Other food groups and endothelial dysfunction and low-grade inflammation

**Vegetable, fruit, fish** and **dairy product** consumption were neither associated with endothelial dysfunction (Supplemental Table 1) nor with low-grade inflammation (Supplemental Table 1).

### Results according to the presence or absence of clinical disease

The consumption of red wine was inversely associated with the low-grade inflammation circulation biomarker score in participants with clinical disease (Supplemental Table 2; P-interaction: 0.09). In addition, the results for dairy products showed that the consumption of dairy products was inversely associated with the endothelial dysfunction biomarker score in participants with clinical disease (Supplemental Table 2: P-interaction: 0.07).

As the latter result appeared in contrast to our hypothesis, we repeated stratified analyses for the following components of total dairy products: cheese, low (<2.0%)- and high (≥2.0%)-fat dairy products. Although not statistically significant, the results showed an inverse association for low-fat dairy production consumption in participants with clinical disease as opposed to a positive association for high-fat dairy products in participants without clinical disease (Supplemental Table 2).

### Additional analyses

**Fig. 1:**
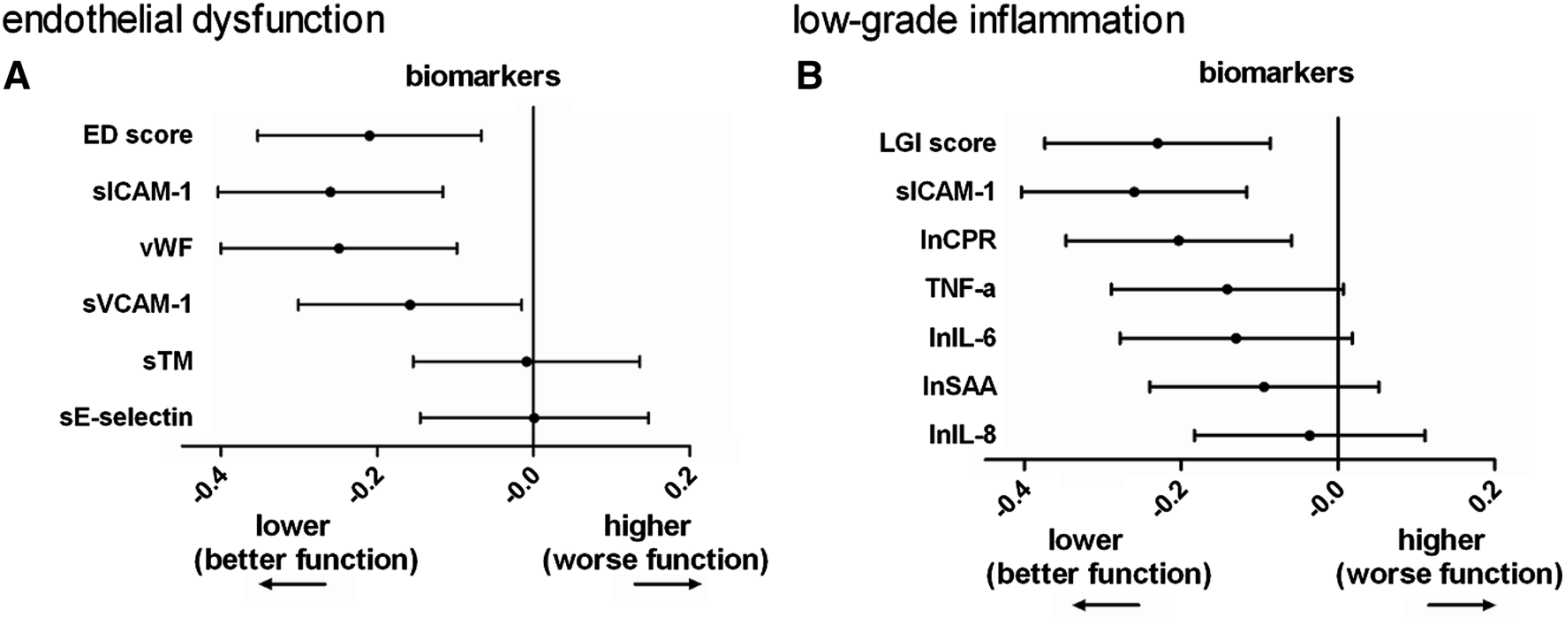
Endothelial dysfunction biomarkers (**a**) and low-grade inflammation biomarkers (**b**) for red wine consumers, compared to nonconsumers. *Dots* show scores and individual biomarker associations adjusted conform models 2, with whiskers indicating their 95% confidence intervals. Higher biomarkers indicate worse function and more inflammation. *ED* endothelial dysfunction biomarker score; *vWf* von Willebrand factor; *sICAM-1* soluble intercellular adhesion molecule 1; *sVCAM-1* soluble vascular cell adhesion molecule 1; *sTM* soluble thrombomodulin; *sE-selectin* soluble endothelial selection; *LGI* low-grade inflammation biomarker score; *TNF-a* tumour necrosis factor alpha; *ln* natural log normalized; *CRP* C-reactive protein; *IL-6* interleukin-6; *SAA* serum amyloid A; *IL-8* interleukin 8

Results for individual biomarkers (Fig. 1), other potential confounders, alternative categorization of alcohol and red wine consumption, other types of alcoholic beverages and accounting for missing data have been addressed in the online supplement.

## Discussion

The present study builds on previous reports, which focused on diet scores or single food components [3–8], by investigation of each of the independent associations between fruit, vegetables, fish, dairy products, alcohol and red wine consumption and endothelial dysfunction (determined by a set of five plasma biomarkers and FMD), in addition to low-grade inflammation (determined by a set of six plasma biomarkers), within one population-based cohort. The study had two main findings. Firstly, after adjustment for potential confounders, both alcohol and red wine consumption were associated with a lower endothelial dysfunction biomarker score and a greater FMD. The associations for FMD were, however, not statistically significant. Importantly, these results were independent of each of the other food components of a healthy diet. Secondly, after adjustment for potential confounders, none of the food groups showed an association with the low-grade inflammation biomarker score, except for the consumption of red wine. Red wine consumption was associated with a lower low-grade inflammation biomarker score. This association was independent of each of the other food components of a healthy diet and was strongest in participants with clinical disease.

Healthy dietary components may improve endothelial dysfunction and low-grade inflammation through several potential mechanisms, such as inhibition of intracellular signalling pathways that activate the inflammatory response, reduction of oxidative stress, modulation of cell membrane stability, modulation of endothelial nitric oxide synthase activity, modulation of the eicosanoid pathway by alteration of prostaglandin synthesis and modulation of endothelial-derived hyperpolarizing factor, which may influence vascular smooth muscle cell tone [13, 31–33]. The present results expand previous knowledge on the associations between diet and endothelial dysfunction and low-grade inflammation [3–11] as it showed that in particular alcohol-containing beverage consumption (i.e. alcohol and red wine) was independently associated with less endothelial dysfunction and less low-grade inflammation. In addition, the observation that the association for red wine and low-grade inflammation was stronger in participants with diabetes and CVD may shed some new light upon the dietary recommendations with regard to the consumption of alcohol and red wine in these patients [29].

The vascular endothelium has many functions in a heterogeneous way throughout the vasculature [22]. The extensive array of endothelium-derived biomarkers and FMD (a measure of endothelium-dependent vasodilation) capture several dimensions of endothelial dysfunction [22]. Indeed, both higher concentrations of the biomarkers and a smaller FMD response are associated with (incident) CVD [34, 35]. The present results for alcohol and red wine showed that, in particular, high alcohol consumers and red wine consumers had a lower endothelial dysfunction biomarker score and greater FMD, which have been associated with less CVD. The results for FMD were, however, not statistically significant. This might be explained by the fact that 158 participants missed FMD data due to a greater body mass index [15], which by itself is associated with a less healthy diet [36] and a smaller FMD response [15]. Additionally, the fact that associations for FMD were investigated in 643 individuals versus 738 individuals for biomarker scores may have led to underestimation of the associations for FMD as compared to those of biomarker scores. Alternatively, the results suggest differential effects of dietary compounds on individual dimensions of endothelial dysfunction, as suggested by previous studies [6, 10, 13].

Additionally, it is unclear to what extent alcohol itself (i.e. ethanol) or any additional nutrient components such as polyphenols and flavonoids may determine the association between alcohol and endothelial dysfunction and low-grade inflammation [13]. In the present study, the association between alcohol consumption and the endothelial dysfunction biomarker score was attenuated after adjustment for red wine, while the associations between red wine and the endothelial dysfunction and low-grade inflammation biomarker scores did not materially change after adjustment for alcohol consumption. This suggests that red wine itself, rich in polyphenols and flavonoids, determines its association with lower adhesion molecules and inflammatory mediators (i.e. biomarkers) independent of its alcohol content [12], while an association between alcohol and biomarkers that is independent from red wine cannot be excluded.

A role for the other food components of a healthy diet on endothelial dysfunction and low-grade inflammation was less clear. In particular, we hypothesized that lower total dairy product consumption was favourably associated with low-grade inflammation. However, the result for total dairy product consumption in participants with clinical disease appeared to oppose our hypothesis and we cannot fully rule out the play of chance. Nevertheless, additional analyses with components of total dairy products showed that more low-fat dairy product consumption was associated with lower biomarkers of endothelial dysfunction in diabetes and CVD, whereas more high-fat dairy product consumption was associated with higher biomarkers of endothelial dysfunction in participants free of clinical disease. This observation might explain how high-fat, but not low-fat, dairy product consumption was associated with CVD mortality [17, 30]. It is however important to realize that these results, despite their association as given by the regression coefficients, were not statistically significant and should be interpreted with caution.

Limitations of the study may have affected our results. Firstly, although we hypothesized that more vegetable, fruit and fish consumption were favourably associated with endothelial dysfunction and low-grade inflammation [1], we cannot exclude that the variation in consumption between persons was too small to reveal any associations between these food groups and endothelial dysfunction and low-grade inflammation. Alternatively, effects of (any of) these food groups might primarily be seen earlier in life [27]. Thirdly, it is likely that participants with an unhealthy diet, worse endothelial dysfunction and worse low-grade inflammation have died before the start of the study causing a survival bias that could have led to an underestimation of the reported associations [1]. Furthermore, any inferences towards causality should be interpreted with caution as the data are cross-sectional. Finally, intrinsic to an overall biomarker score is the assumption that each biomarker in the score carries similar weight. Consequently, this approach may not optimally reflect pathophysiology as it is unknown if the relationship between food intake and circulating biomarkers as a proxy for cardiovascular disease is similar for each circulating biomarker.

In conclusion, alcohol and red wine consumption were associated with lower biomarkers of endothelial dysfunction and red wine consumption was associated with lower biomarkers of low-grade inflammation, whereas results for vegetable, fruit, fish and dairy product consumption were less clear. Importantly, these results were independent of vegetable, fruit, fish and dairy product consumption. This suggests that food components of a healthy diet, and in particular alcohol-containing beverage consumption, may favourably influence endothelial dysfunction and low-grade inflammation.

## Electronic supplementary material

Below is the link to the electronic supplementary material.

Supplementary material 1 (DOCX 22 KB)
