# Supplementary material for: Alcohol and red wine consumption, but not fruit, vegetables, fish or dairy products, are associated with less endothelial dysfunction and less low-grade inflammation: the Hoorn Study
Source: Eur J Nutr. 2017 Mar 27;57(4):1409–19. doi: 10.1007/s00394-017-1420-4 (PMC5959974; doi:10.1007/s00394-017-1420-4)
Supplement: Supplementary file 1 — Supplementary material 1 (DOCX 22 KB) [file 394_2017_1420_MOESM1_ESM.docx]

**Additional analyses**

Additional adjustment for total and HDL cholesterol, systolic blood pressure, the use of lipid-lowering and/or anti-hypertensive medication, in addition to eGFR, did not materially change the results (data not shown)

When we re-analyzed the data with adjustment for both current and previous smokers (separate dummy variables) the results did not materially change (data not shown).

When we re-analyzed the data for red wine and the individual biomarkers of the endothelial dysfunction biomarker score, the results showed inverse associations for each individual biomarker, except for sE-selectin (Figure 1A). When we re-analyzed the data for red wine and the individual biomarkers of the low-grade inflammation biomarker score, the results showed inverse associations for each individual biomarker (Figure 1B). Re-analyses of the data, while taking sICAM-1 out of each of the biomarker scores, did not materially change the results (data not shown).

Results did not materially change when trends were tested using the median intake per category of alcohol consumption. Alternative analyses with red wine consumers divided into moderate consumers (red wine intake: >0 and ≤75 mL/d) and high consumers (red wine intake: >75 mL/d) gave similar results (P-trend = 0.034 for the endothelial dysfunction biomarker score and P-trend = 0.028 for the low-grade inflammation biomarker score, data not shown). The Pearson’s correlation coefficient between alcohol and red wine was r=0.34. When we performed mutual adjustments for red wine and alcohol with categorical data only the results did not materially change.

When we re-analyzed the models replacing red wine consumers for beer, liquor or white wine consumers, we did not find any statistically significant associations with the endothelial dysfunction biomarker score, FMD or the low-grade inflammation biomarker score (Supplemental Table 3).

Missing covariate data were replaced by the population’s mean. When we restricted the analyses to the subset of participants with full covariate data, re-analyses of the data gave similar results (data not shown).

Models for FMD without adjustment for baseline diameter gave similar results. When we restricted the analyses for FMD to the subset of participants without missing post-occlusion diameters, re-analyses of the data also gave similar results (data not shown).

**Supplemental Table 1.** Association between tertiles of vegetable, fruit, fish and dairy product consumption and endothelial dysfunction and low-grade inflammation

|  | **Endothelial dysfunction** | | | | |  | **Low-grade inflammation** | |
| --- | --- | --- | --- | --- | --- | --- | --- | --- |
|  | biomarker score | |  | flow-mediated vasodilation | |  | biomarker score | |
|  | β (95%CI) P-value | P-trend |  | β (95%CI) P-value | P-trend |  | β (95%CI) P-value | P-trend |
| (A) Vegetable |  |  |  |  |  |  |  |  |
| low | - |  |  | - |  |  | - |  |
| moderate | -0.07 (-0.24;0.10) 0.406 |  |  | 0.06 (-0.12;0.23) 0.544 |  |  | -0.07 (-0.24;0.10) 0.402 |  |
| high | -0.09 (-0.26;0.08) 0.308 | 0.306 |  | 0.06 (-0.12;0.25) 0.493 | 0.492 |  | -0.04 (-0.21;0.14) 0.669 | 0.666 |
| (B) Fruit |  |  |  |  |  |  |  |  |
| low | - |  |  | - |  |  | - |  |
| moderate | 0.06 (-0.11;0.23) 0.490 |  |  | 0.11 (-0.08;0.29) 0.252 |  |  | -0.08 (-0.26;0.09) 0.341 |  |
| high | 0.02 (-0.15;0.20) 0.790 | 0.798 |  | 0.12 (-0.07;0.31) 0.223 | 0.226 |  | -0.05 (-0.23;0.13) 0.563 | 0.572 |
| (C) Fish |  |  |  |  |  |  |  |  |
| low | - |  |  | - |  |  | - |  |
| moderate | 0.04 (-0.13;0.21) 0.662 |  |  | -0.11 (-0.29;0.07) 0.227 |  |  | 0.06 (-0.11;0.23) 0.489 |  |
| high | -0.04 (-0.21;0.13) 0.617 | 0.608 |  | 0.02 (-0.16;0.20) 0.836 | 0.816 |  | -0.03 (-0.21;0.14) 0.706 | 0.695 |
| (D) Dairy products |  |  |  |  |  |  |  |  |
| low | - |  |  | - |  |  | - |  |
| moderate | -0.01 (-0.18;0.16) 0.910 |  |  | -0.14 (-0.32;0.04) 0.125 |  |  | 0.17 (-0.01;0.34) 0.058 |  |
| high | -0.13 (-0.30;0.04) 0.132 | 0.132 |  | -0.02 (-0.20;0.17) 0.860 | 0.869 |  | -0.02 (-0.20;0.15) 0.799 | 0.800 |

Data are standardized regression coefficients, with their 95% confidence intervals, indicating the difference in the endothelial dysfunction biomarker score, flow-mediated vasodilation (FMD) and the low-grade inflammation biomarker score (all in SD) between (A) tertiles of vegetable consumption, with moderate [median (IQR) 122 (113-131) g/d] and high consumers [173 (157-195) g/d] compared to low consumers [79 (63-92) g/d (-, reference group)]; (B) tertiles of fruit consumption, with moderate [median (IQR) 266 (242-298) g/d] and high consumers [423 (385-511) g/d] compared to low consumers [126 (83-153) g/d (-, reference group)]; (C) tertiles of fish consumption, with moderate [9.8 (7.5-14.0) g/d] and high consumers [18.9 (16.8-31.2) g/d] compared to low consumers [0.8 (0-2.7) g/d (-, reference group)]; and (D) tertiles of dairy product consumption, with moderate [410 (381-450) g/d] and high consumers [644 (589-786) g/d] compared to low consumers [216 (151-270) g/d (-, reference group)]; with P-values and P-trend by linear regression analyses.

Analyses on biomarker scores include 738 participants and higher values indicate worse function.

Analyses on FMD include 643 participants and lower values indicate worse function; to calculate peak diameter change in millimeters multiply by 0.167 (SD)

Models adjusted for sex, age, glucose metabolism status, energy intake, body mass index, current smoking, prior cardiovascular disease, educational level, physical activity, and alcohol (A, B, C and D), vegetable (B, C and D only), fruit (A, C and D only) fish (A, B and D only) and dairy product (A, B and C only) consumption and for FMD additionally for baseline diameter and flow increase.

**Supplemental Table 2**. Associations between food consumption and endothelial dysfunction and low-grade inflammation biomarker scores, stratified according to the presence of clinical disease.

| Model | | **Low-grade inflammation** | | | | |
| --- | --- | --- | --- | --- | --- | --- |
|  |  | free of clinical disease | |  | presence of clinical disease | |
|  |  | β (95%CI) P-value |  |  | β (95%CI) P-value |  |
|  | **Red wine** |  |  |  |  |  |
| 1 | nonconsumers | - |  |  | - |  |
|  | consumers | -0.050 (-0.299;0.198) 0.689 |  |  | **-0.247 (-0.434;-0.059) 0.010** |  |
|  |  | **Endothelial dysfunction** | | | | |
|  |  | free of clinical disease | |  | presence of clinical disease | |
|  |  | β (95%CI) P-value | P-trend |  | β (95%CI) P-value | P-trend |
|  | **Dairy products** |  |  |  |  |  |
| 2 | low | - |  |  | - |  |
|  | moderate | 0.136 (-0.124;0.396) 0.305 |  |  | **-0.071 (-0.289;0.148) 0.525** |  |
|  | high | 0.052 (-0.205;0.308) 0.693 | 0.692 |  | **-0.250 (-0.480;-0.021) 0.032** | **0.033** |
|  | **Low-fat dairy products** |  |  |  |  |  |
| 2 | low | - |  |  | - |  |
|  | moderate | -0.018 (-0.227;0.241) 0.891 |  |  | -0.103 (-0.324;0.117) 0.357 |  |
|  | high | 0.015 (-0.243;0.274) 0.906 | 0.487 |  | -0.167 (-0.391;0.056) 0.141 | 0.344 |
|  | **High-fat dairy products** |  |  |  |  |  |
| 2 | low | - |  |  | - |  |
|  | moderate | **0.299 (-0.036;0.562) 0.026** |  |  | 0.095 (-0.124;0.314) 0.396 |  |
|  | high | 0.213 (-0.061;0.487) 0.128 | 0.280 |  | -0.175 (-0.407;0.056) 0.137 | 0.169 |
|  | **Cheese** |  |  |  |  |  |
| 2 | low | - |  |  | - |  |
|  | moderate | 0.028 (-0.236;0.291) 0.836 |  |  | -0.050 (-0.269;0.169)0.653 |  |
|  | high | -0.055 (-0.314;0.204) 0.677 | 0.991 |  | -0.113 (-0.341;0.115) 0.329 | 0.836 |

Data are standardized regression coefficients, with their 95% confidence intervals, indicating the difference in the endothelial dysfunction biomarker score, and the low-grade inflammation biomarker score (all in SD) between red wine consumers [median (IQR) 28.6 (5.5-71.4) dL/d] and nonconsumers (-, reference group) and between tertiles of dairy product consumption, with moderate [410 (381-450) g/d] and high consumers [644 (589-786) g/d] compared to low consumers [216 (151-270) g/d (-, reference group)] according to strata of clinical disease; with P-values and P-trend by linear regression analyses; n = 507 participants with clinical disease: either CVD (n = 238), diabetes mellitus type 2 (n = 110), or both (n = 159); n = 231 participants without clinical disease.

model 1: adjusted for sex, age, glucose metabolism status, energy intake, body mass index, current smoking, prior cardiovascular disease, educational level, physical activity, and vegetable, fruit, fish and dairy product consumption.

models 2: adjusted for sex, age, glucose metabolism status, energy intake, body mass index, current smoking, prior cardiovascular disease, educational level, physical activity, and vegetable, fruit, fish and alcohol consumption.

**Supplemental Table 3**. Associations between categories of alcohol and red wine consumption and endothelial dysfunction and low-grade inflammation

| model | | **Endothelial dysfunction** | | | | |  | **Low-grade inflammation** | |
| --- | --- | --- | --- | --- | --- | --- | --- | --- | --- |
|  |  | biomarker score | |  | flow-mediated vasodilation | |  | biomarker score | |
|  |  | β (95%CI) P-value |  |  | β (95%CI) P-value |  |  | β (95%CI) P-value |  |
|  | **Beer** |  |  |  |  |  |  |  |  |
| 1 | nonconsumers | - |  |  | - |  |  | - |  |
|  | consumers | -0.01 (-0.18;0.16) 0.903 |  |  | -0.12 (-0.30;0.05) 0.163 |  |  | -0.11 (-0.28;0.06) 0.215 |  |
| 2 | nonconsumers | - |  |  | - |  |  | - |  |
|  | consumers | 0.01 (-0.16;0.18) 0.897 |  |  | -0.13 (-0.31;0.04) 0.132 |  |  | -0.09 (-0.26;0.08) 0.300 |  |
| 3 | nonconsumers | - |  |  | - |  |  | - |  |
|  | consumers | 0.01 (-0.16;0.18) 0.906 |  |  | -0.14 (-0.31;0.04) 0.118 |  |  | -0.09 (-0.26;0.08) 0.298 |  |
|  | **Liquor** |  |  |  |  |  |  |  |  |
| 1 | nonconsumers | - |  |  | - |  |  | - |  |
|  | consumers | -0.11 (-0.25;0.04) 0.144 |  |  | 0.04 (-0.12;0.19) 0.655 |  |  | 0.07 (-0.08;0.22) 0.350 |  |
| 2 | nonconsumers | - |  |  | - |  |  | - |  |
|  | consumers | -0.12 (-0.26;0.02) 0.103 |  |  | 0.05 (-0.11;0.20) 0.547 |  |  | 0.06 (-0.08;0.20) 0.414 |  |
| 3 | nonconsumers | - |  |  | - |  |  | - |  |
|  | consumers | -0.12 (-0.26;0.03) 0.114 |  |  | 0.04 (-0.11;0.19) 0.606 |  |  | 0.06 (-0.08;0.21) 0.387 |  |
|  | **White wine** |  |  |  |  |  |  |  |  |
| 1 | nonconsumers | - |  |  | - |  |  | - |  |
|  | consumers | -0.11 (-0.25;0.03) 0.133 |  |  | 0.09 (-0.06;0.23) 0.254 |  |  | -0.10 (-0.24;0.04) 0.173 |  |
| 2 | nonconsumers | - |  |  | - |  |  | - |  |
|  | consumers | -0.06 (-0.20;0.08) 0.403 |  |  | 0.06 (-0.09;0.21) 0.413 |  |  | -0.05 (-0.19;0.09) 0.483 |  |
| 3 | nonconsumers | - |  |  | - |  |  | - |  |
|  | consumers | -0.06 (-0.20;0.08) 0.430 |  |  | 0.06 (-0.09;0.21) 0.453 |  |  | -0.05 (-0.19;0.10) 0.516 |  |

Data are standardized regression coefficients, with their 95% confidence intervals, indicating the difference in the endothelial dysfunction biomarker score, flow-mediated vasodilation (FMD) and the low-grade inflammation biomarker score (all in SD) between beer consumers [median (IQR) 57.1 (11.0-171.4) dL/d] and nonconsumers of beer, between liquor consumers [median (IQR) 21.4 (5.0-75.0) dL/d] and nonconsumers of liquor, and between white wine consumers [median (IQR) 6.7 (2.7-28.6) dL/d] and nonconsumers of white wine. (-, reference group); with P-values.

Of 801 individuals 302 consumed beer, 346 consumed white wine and 444 consumed liquor.

Analyses on biomarker scores include 738 participants and higher values indicate worse function.

Analyses on FMD include 643 participants and lower values indicate worse function; to calculate peak diameter change in millimeters multiply by 0.167 (SD).

model 1: adjusted for sex, age, glucose metabolism status, energy intake, and for FMD additionally for baseline diameter and flow increase.

model 2: model 1 additionally adjusted for body mass index, current smoking, prior cardiovascular disease, educational level and physical activity.

model 3: model 2 additionally adjusted for vegetable, fruit, fish and dairy product consumption.
